# Prevalence and risk factors to HIV-infection amongst health care workers within public and private health facilities in Cameroon

**DOI:** 10.11604/pamj.2018.29.158.14073

**Published:** 2018-03-19

**Authors:** Irénée Kammogne Domkam, Nelson Sonela, Nelly Kamgaing, Patrice Soh Takam, Luc-Christian Gwom, Thomas Michel Anana Betilene, Joseph Fokam, Serge Clotaire Billong, Laure Vartan Moukam, Tatiana Mossus Etounou, Christine Sara Minka Minka, Alexis Ndjolo

**Affiliations:** 1 Chantal Biya International Reference Center for Research on HIV/AIDS Prevention and Management (CIRCB), Yaounde, Cameroon; 2 University of Yaounde I, Yaounde, Cameroon; 3 Association Camerounaise pour le Marketing Social (ACMS), Cameroon; 4 National AIDS Control Committee, Yaounde, Cameroon; 5 Ministry of Public Health, Cameroon

**Keywords:** HIV, health care worker, prevalence

## Abstract

**Introduction:**

This study aimed at assessing the prevalence of Human Immunodeficiency Virus (HIV) among health care workers (HCWs) and to evaluate some risks factors for HCWs.

**Methods:**

We conducted a cross sectional study amongst HCWs in public and private healthcare facilities within seven regions amongst the 10 found in Cameroon. We collected data from 446 HCWs within 150 healthcare facilities. We used questionnaires for interviews and biological sampling for HIV test.

**Results:**

HIV prevalence was 2.61% (95% CI: 1.32% - 4.61%) regardless of gender and age. HCWs in private health facilities were more infected compared to those in public health facilities 5.00% vs 1.40% (p = 0.028); OR = 3.7 (95% CI: 1.01-12.90). HCWs who had never screened for HIV had a high risk of being infected OR = 7.05 (95% CI: 2.05-24.47). 44.62% of HCWs reported to have been victim of an Accidental Exposure to Blood (AEB). Amongst them, 45.80% in public HF versus 32.1% in private HF reported to have received an HIV screening and Post Exposure Prophylaxis following this incident. 4.20% of HCW victim of AEB were HIV positive, and 36.40% of HCWs had appropriate capacity training for HIV patient care.

**Conclusion:**

Though the HIV prevalence in HCWs is lower than in the general population 2.61% vs 4.3%, there is a high risk of infection as we observed a relatively high percentage of AEB amongst HCWs with an HIV prevalence of 4.20%. There is thus, a need in strengthening the capacity and provide psychosocial support to HCWs.

## Introduction

According to the Joint United Nations Program on HIV/AIDS (UNAIDS), 36.7 (32.2 to 38.8) million people were living with HIV in the world in 2015 [1]. Amongst these People Living with HIV (PLHIV), more than 69% live in the Sub-Saharan Africa where studies demonstrate that one in twenty adults live with HIV [2]. The Demographic and Health Survey (DHS) of 2011 in Cameroon estimated the prevalence of HIV to be around 4.3% [3]. This prevalence ranks Cameroon among the five countries of the East and Central African region where HIV prevalence is highest [4]. Studies have evaluated the prevalence of HIV amongst Health Care Workers (HCWs) and reported a relatively high prevalence of HIV and AIDS amongst HCWs 15.7% [95% confidence interval (CI): [12.2-19.9%] employed in the public and private health facilities [5]. These findings make HCWs a special population to be considered in the fight against HIV and AIDS as they are in contact with patients and constantly subjected to occupational exposures in the course of their duty. There is thus, need for special consideration as far as HCWs are concerned in the implementation of HIV programs [6]. With progress achieved in the management of HIV and the relative availability as well as the use of Antiretroviral (ARV), there has been a great increased in the life expectancy of People Living with HIV (PLHIV) resulting in an increase in HIV-related morbidity. This has resulted in an increase in workload, an increase in demand for healthcare services and subsequently increased risk of nosocomial and occupational infections for HCWs. In fact, the scarcity of HCWs, stressful schedules and heavy workloads as well as increased number of patients have resulted in an increase risk occupational exposure to blood and acquired blood-borne infections [7]. In South Africa, a study on the impact of HIV found that HIV prevalence amongst HCWs was 15.7% and 28% among patients attending the same health facilities. This study also showed a link between the prevalence of HIV amongst patients and the prevalence observed amongst HCWs [8].

Occupational exposures to blood borne infections represent a major risk factor for HCWs. It is reported that, HCWs incur about 2 million needle stick injuries (NSIs) per year that result in infections with hepatitis B and C and HIV. The World Health Organization estimates that, the global burden of disease from occupational exposure is around 40% of the hepatitis B and C infections and 2.5% of the HIV [9,10]. It has been demonstrated that, the level of risk to occupational exposure greatly depends on the number of patients, experience and the precautions observed by HCWs during health care services delivery to patients [8]. Unfortunately, health care professionals are most negligent as far as their own health is concerned despite being exposed to high risk of contracting various infections and the risk of also becoming victims of lifestyle diseases. This attitude amongst HCWs is bolstered by an uncooperative unwillingness to get tested by HCWs and high rates of AIDS-related morbidity and mortality amongst HCWs. Studies have revealed that there is a low uptake of HIV screening and access to HIV care amongst HCWs.

In the Sub-Saharan Africa, where HIV and AIDS is most prevalent PLHIV occupied around 50% of hospital beds [8,11,12]. Due to this heavy work pressure, organizations and services that provide healthcare services and support to patients have difficulties in achieving an efficient management [4]. This situation is exacerbated by the fact that, the health sector in countries most affected by the HIV and AIDS pandemic is faced with limited human and financial resources [9]. The USAID in one of its reports pointed out that many countries in the sub-Saharan Africa suffer from a scarcity of trained HCWs of almost all socio-professional levels primarily due to morbidity and mortality as a result of HIV and AIDS [7]. Considering the relatively high prevalence of HIV in Cameroon, the risk for the hospital system in Cameroon and the need to define appropriate policies to improve healthcare for PLHIV, we conducted a cross-sectional study with the aim of evaluating the prevalence of HIV and AIDS among HCWs in public and private health facilities and to evaluate some risk factors for HIV infection amongst HCWs in Cameroon. Indeed, the measure and understanding of the professional and social damage of HIV within the health community (HCWs and patients) not only would allow for the better protection of health personnel, but also a more appropriate deployment of human, material and financial resources allocated to the fight against HIV.

## Methods

### Study sites and population

This study was conducted in 7 regions of Cameroon amongst which the Central, Eastern, Littoral, West, Northwest and Southwest regions. The regions of Adamawa, North and Far North were not included because of the political insecurity at the moment of sample collection. The observational units for this study consisted mainly of health care facilities from the 7 surveying regions cited above. The sampling frame consisted of a list of all private and public health facilities provided by the Ministry of Public Health. In order to provide a robust estimate for the study, a sample of 150 health facilities were considered.

### Procedures

The study was a cross-sectional voluntary and anonymous study conducted amongst 446 health care workers (HCWs). Data was collected using a comprehensive questionnaire administered in a face-to-face interview between the research staff and each participant. Blood sample was collected from each participant for HIV screening. Prior to the start of the study, the questionnaires were pretested and validated. Data collection was conducted within the months of January and February 2015.

Were enrolled in the study HCWs from all socio-professional cadre (Medical Doctors, the Nursing staff, Medical-Laboratory technicians, etc.) in attendance at work on data collection days, aged 21 years and above, willing to take an HIV screening and give a signed written informed consent. Each participant enrolled in the study was screened for HIV using a modified National HIV screening algorithm. Participants were first screened with ALERE Determine HIV-1/2 Kit rapid diagnostic test. Positive cases were confirmed with HIV 1/2 Bispot ImmunoComb. Western blot assay was use as a tiebreaker in this study. Laboratory results were recorded in a laboratory logbook, which were then transcribed on each participant’s corresponding questionnaire by a supervisor in charge of the sampling site.

Data was entered on EpiData software, cleaned and analyzed with R.3.1.3. Results are shown as proportions with a 95% Confidence Interval (CI) for categorical variables and means±SD for quantitative variables. The chi-square test was used to assess the association between the type of HFs (Private, Public) vs attitudes of HCWs with respect to HIV screening (History of screening, accidental exposure to blood, circumstance of screening); the serological status (positive, negative) vs HCW characteristics (gender, age, professional categories, screening history, time since the last screening and type of HFs). Univariate logistic regression was then used to quantify the relationship between the HIV serological status and HCW characteristics expressed as ORs 95% CI. The differences between the results were considered statistically significant at p-value = 0.05 threshold.

### Ethical considerations

This study received ethical approval from the Cameroonian National Ethics Committee for Research on Human Subjects, Ethical Clearance number No. 2015/06/606/EC/CNERSH/SP. All participants signed the informed consent form.

## Results

Data was collected in 150 health facilities (HF) that served as the sampling frame for this study. 65% of these HF were public health facilities versus 35% private health facilities. In the public sector, 23% of the health facilities were District Hospitals, 18% District Health Centers and 59% Integrated Health Centers.

### Baseline information on Health care workers (HCWs)

A total of 446 health care workers (HCWs) were recruited and enrolled in the study. A critical look at the Table 1. summarizing the individual characteristics of participants reveals that 72.65% of those who agreed to participate in the study were mostly female HCWs. Furthermore, the average age of participants was significantly different depending on whether they were men or women. Data showed that the mean age was much higher for men 36.43 years [24.65, 42.83] than for women 33.74 years [26.1, 46.76] P < 0.05. With regards to the socio-professional category, medical doctors, nurses, laboratory, midwives and ward-attendants were amongst those recruited. Nurses were the most represented (36.1%), while Ward-attendants were the less represented 20 (4.5%). Analyses with respect to the number of sexual partners showed that 72% HCWs lived with only one sexual partner. Out of this 72%, 73.8% were women, while 67.2% were men. Multiple sexual partnership on the other hand was practiced in about 5.4% of enrolled HCWs out of which 2.2% women and 13.9% men. There was a total of 101 HCWs (22.6%) who reported to be living without a partner at the time of sample collection amongst which 24.1% women and 18.9% are men.

**Table 1:** Distribution of HCWs sample by individual characteristics

	Female	Male	Total
**Socio-professional Categories**			
Medical Doctors	101 (31.2%)	16 (13.1%)	117 (26.2%)
Nurses	113 (34.9%)	48 (39.3%)	161 (36.1%)
Midwives	16 (4.9%)	8 (6.6%)	24 (5.4%)
Laboratory personnel	79 (24.4%)	45 (36.9%)	124 (27.8%)
Ward-Aids	15 (4.6%)	5 (4.1%)	20 (4.5%)
**Age**			
**Mean±Standard Deviation**	33.74±9.09	36.43±10.33	
**Number of partners**			
One partner	239 (73.8%)	82 (67.2%)	321 (72%)
Many Partners	7 (2.2%)	17 (13.9%)	24 (5.4%)
Without Partner	78 (24.1%)	23 (18.9%)	101 (22.6%)
TOTAL	324 (72.65%)	122 (27.35%)	446 (100%)

### HIV screening test amongst HCWs

This section assessed the attitudes of HCWs towards HIV screening according to three criteria: (i) the history of screening, (ii) the time elapsed since the last screening and (iii) the reasons which led to the last HIV screening test. An analysis of the attitude of HCWs with respect to HIV screening categorized by the type of health facility is represented in Table 2. Data showed that, 94% of HCWs in public and private health facilities had already been screened for HIV at the time of the study. With regards to the circumstances which led to testing, the majority of participants reported to have taken an HIV screening during a voluntary screening, that is 55% in public against 60% in private Health facilities (HF) (P-value = 0.7). Analysis of the circumstances which led towards the last screening indicates that more than half of HCWs (57.2%) volunteered to do the screening. This attitude was observed more in men (67.2%) than in women (53.4%). Furthermore, 16.3% of participants took their last HIV screening during a medical consultation (18.9% for women against 9.5% for men). About one in 10 (9.5%) did their last screening during outreach campaign. 8.3% of the participants said they did their screening for other reasons while 4.7% and 4% respectively did so for professional reasons and after an AEB. A total of 24 (5.4%) HCWs had never been tested for HIV before. Of these, 10 (42%) mentioned fear of the result while 14 (58%) said they were not concerned.

**Table 2:** HIV screening test according to the type of health facility

	Total	Public Health facility	Private Health facility	P-value
	446	291	155	
**HCWs who have done HIV screening**				
*Yes*	422	94.80%	94.20%	0.771
*No*	24	5.20%	5.80%	
**Circumstances leading to last HIV screening**				
Voluntary screen	242	55.60%	60.30%	0.705
Outreach Campaigns	40	10.10%	8.20%	
After Accident Blood exposure	17	4.70%	2.70%	
Professional Obligation	20	4.00%	6.20%	
Medical Consultation	69	17.00%	15.10%	

Screening history revealed that, only 9.5% of the HCWs did their last test within the last twelve months. On average, the time elapsed since the last HIV screening was about 16 months in women. However, some claimed to have been screened less than a year ago, while others claimed to have been screened 10 years ago. On the other hand, the average time since the last HIV screening in men was 1.72 year. Though some claimed to have been tested 16 years ago.

### HIV prevalence and mortality among HCWs

HIV prevalence among HCWs and analysis of factors associated with HIV status are shown in Table 3. As indicated in this Table, the data from this study revealed that the prevalence of HIV amongst HCWs is 2.61% (1.3% - 4.6%). Analyses revealed that there is no association between the obtained prevalence of HIV and sex, age, socio-professional category and number of partners. But on the other hand, when considering the type of health facility, HCWs from private health facilities were more affected than those from the public health facilities, OR = 3.71 (1.0 to 12.9). The screening history was also very important. Indeed, those who had never been tested for HIV had a very high probability of being infected OR = 11.8 (3.18 to 43.88). This trend was the same when we took into consideration the period since the last test. Thus, the HIV prevalence was lower amongst those who had their HIV screening between 12 and 24, and highest among those who made their test more than two years ago, OR = 0.23 (0.071 to 0.82). The prevalence of HIV, although not significant was observed to be higher amongst HCWs who claimed to have already had an AEB 4.2%, versus 1.3% for those who have never been victims of an AEB. P-value = 0.07

**Table 3:** HIV prevalence among health care workers (HCWs)

	Total	N	percentage	CI 95%	P-Value	OR 95%
	421	11	2.61%	1.3 - 4.6		
**Age group**						
*18-24 years*	50	1	2.0%	0.05 - 10.6	0.95	
*25-35 years*	215	6	2.8%	1 - 5.9		
*>35 years*	156	4	2.6%	0.7 - 6.4		
**Sex**						
*Female*	303	9	3.0%	1.3 - 5.5	0.468
*Male*	117	2	1.7%	0.2 - 6	
**Number of sexual Partners**						
*One*sexual *Partner*	303	7	2.3%	0.9 - 4.7	0.767	
*Many*sexual *Partner*	22	1	4.5%	0.1 - 22.8		
*No*sexual *Partner*	96	3	3.1%	0.6 - 8.8		
**Professional Category**						
*Medical doctor*	22	0	0.0%		0.769	
*Nurse*	143	5	3.5%	1.1 - 7.9		
*Midwife*	19	1	5.3%	0.1 - 26		
*Laboratory technician*	120	3	2.5%	0.5 - 7.1		
*Ward-attendant*	111	2	1.8%	0.2 -6.3		
**Have you already been screened for HIV**						
*Yes*	400	7	1.8%	0.7 - 3.5	0.000	11.8 (3.18-43.88)
*No*	23	4	17.4%	4.9 - 38.7		
**Type of Health facility**						
*Public*	284	4	1.4%	0.3 - 3.5	0.028	3.71 (1.0-12.9)
*Private*	139	7	5.0%	2 - 10		
**Time elapsed since last screening**							
*> 24 month*	87	6	6.9%	2.5 - 14.4	0.017	0.23 (0.071-0.82)
*12-24 months*	288	5	1.7%	0.5 - 4		
*< 12months ©*	41	0	0.0%			

N = Number of positive cases; P = Prevalence;

^+^Values obtained from comparison tests should be considered with caution given the small numbers of positive cases.

© This group of health personnel was excluded from the analysis, as there are no positive cases within for those who made their HIV screening test less than 12 months ago.

### Health care workers and accidental exposure to blood

To evaluate the rate of AEB amongst HCWs. Data was collected based on the declaration made by the HCWs in the various health facilities visited. In total, 199 (44.62%) HCWs reported to have been victims of an AEB. They were almost equally distributed that is 44.1% in the public health facilities versus 45.8% in private health facilities. From HCWs who declared to be victims of AEB. 45.70% HCWs received an HIV care after the accident. However, a comparative analysis revealed that, public HCWs significantly benefited from HIV support care (Screening and Post-exposure prophylaxis (PEP)) after an AEB compared to those from the private health facilities, that is, 46.2% against 32.1% (P = 0.042).

### Availability of personal protective equipment (PPE)

Personal protective equipment is important to ensure the safety of staff and patients as well as in reducing nosocomial infections amongst health care workers. The HCWs interviewed declared that over 80% of all personal protective equipment were available in the hospital. But on the other hand, they declared that the use of these PPE by all the HCWs was poor. It should be noted that the private health facilities possessed more personal protective equipment than do the public health facilities. Gloves were available in 87.6% public health facilities against 94.2% of private health facilities (P = 0.028); we had a non-significant difference for bleach (83.8% and 89% for the public and private respectively) (P = 0.137). We observed significant differences for the availability of alcohol for disinfection and laboratory coats between the public health facilities and private health facilities.

## Discussion

As demonstrated and proven by many studies conducted in Africa and the world at large, the Sub-Saharan African belt bears the brunt of HIV infection [1,2]. Despite the slight reduction in the general prevalence from 5.5% to 4.3%, Cameroon still remains amongst the most affected countries in the world [4]. We found an HIV prevalence of 2.6%, amongst the HCWs. This rate is well below the national prevalence, which is around 4.3%. This low prevalence could be explained by several reasons. One of the most outstanding reason could be that, the majority of HCWs who agreed to be tested were mostly those who already knew their HIV status. A critical look in the data revealed that more than 90% of HCWs had already been screened for HIV.

We observed there was a statistically significant association (p-value = 0.017) between the history of HIV screening and HIV status: those who had never taken the test were more infected than those who had already been tested, and those whose last test had been taken more than two years were most infected than those who were tested within the last two years. We observed an uncooperative attitude from HCWs perceived by the unwillingness to get tested for HIV, observed during sample collection. The same observation was made in a study conducted in South Africa on barriers to testing and counseling HCWs. This study showed that among the reasons given by HCWs, the fear of breach of confidentiality was outstanding (38.9% of respondents) if the screening was conducted in the participants’ health facility as was the case during this study. There was also the fear of stigma that could result from this breach of confidentiality from colleagues (40% of respondents). The majority of HCWs did not want their colleagues to know the results of their HIV screening [13].

Though small, the prevalence of 2.61% (95% CI: 1.31% - 4.61%) obtained within the framework of this study translates a critical situation for HCWs, as far as HIV and AIDS is concern. An application of this prevalence on the total population of staff in the health sector in Cameroon estimated at 38,207 HCWs in 2011 shows that between 496 and 1,758 health care workers live with HIV and AIDS. Moreover, according to estimates by the World Bank, a country with an average HIV prevalence of 5% among its adult population will report a mortality rate between 0.5 and 1% amongst its health care workers [14]. We thus, estimate that around 191-383 HCWs would die per year from HIV and AIDS. It is worth noting that the estimated HIV prevalence among HCWs in this study suffered a selection bias given the reasons outlined above amongst which, fear of breach of confidentiality, unwillingness to get tested for fear of results and most of those who knew their results came for the study. Recent studies have shown that an approach to reduce this bias, knowing the reasons mentioned here would be to set up an HIV screening system where in HCWs would conduct their HIV screening out of their working environment, for instance in their homes. Such is the case of a study to assess the factors associated with the acceptability of HIV self-testing among HCWs in Kenya. This study showed encouraging results, with nearly half of the targeted personnel tested. Although encouraging, the study showed the need for strategies to bring HCWs to follow preliminary briefings. Indeed, only 41% of HCWs attended [15].

HCWs remain a key population to be considered as far as the implementation of HIV and AIDS programs are concern. Morbidity and mortality of HCWs related to occupational exposures has an impact on the workforce, and as a result on access to good health care. HCWs in Sub-Saharan Africa are at high risk of HIV infection from both sexual and occupational exposure [16]. Needle stick injuries have been reported to represent most of occupational exposure. In fact, as estimated in one WHO report, HCWs encountered more than 2million Needle Stick Injuries (NSIs) yearly and the global burden of disease due to HIV is 2.5% [9,10]. In the course of this study we observed that 44.1% of HCWs enrolled have at one point been victims of occupational exposure. Of the 44.1% of HCWs victims of occupational exposure, 4.2% are HIV infected. This prevalence is closed to the national prevalence of 4.3%. Unfortunately, our study revealed that, less than 50% of HCWs victim of occupational exposure received care and post-exposure prophylaxis (PEP).

Although the majority of these personnel did not seek to know their HIV status as was observed by their unwillingness in this study, recent guidelines from WHO/UNAIDS recommend priority access to HIV services for HCWs in order to maintain and better manage these resources [13]. Results of the present study show that, there is a high risk that HCWs do not know their HIV serological status (OR = 11). This ignorance could be a double edge blade as on one hand could be a risk factor for patients or uninfected individuals with regards to HIV transmission and on the other could be a risk factor for HIV positive and immunocompromised HCWs with regards to opportunistic infections (OI) during health care delivery.

It has been shown that the health labor market most especially in developing countries is jeopardized by a global shortage of skilled HCWs of all socio-professional cadres. This is due in part to the migration of skilled HCWs from developing to developed countries due to poor working conditions, low salaries as well as exposures to deadly infectious agents. To make worse the situation there is little capacity strengthening and continuous training on HIV management and related issues for the remaining staff faced with an increase in workload thus, contributing further to illness, injury, dissatisfaction and the desire to migrate [17]. Success in the management of HIV and AIDS patients could be achieved by the appropriate deployment of the available health staff, resources and HIV specific capacity building. We observed that more than 80% of Personal Protective Equipment (PPE) were available but the HCWs needed to be reeducated on the use and importance of PPE. Base on HIV specific capacity building, we identified many gaps with regards to the type of training received by the HCWs on the management of HIV. Overall, less than 50% of staff has been trained for each of the items considered. This is particularly important when we consider issues such as stigma and discrimination against the most at-risk populations (sex workers, MSM and truck drivers, among others) that have high HIV prevalence and are targeted by the National AIDS Control Committee in its strategic plans.

## Conclusion

In the scope of this study we sought to evaluate the prevalence, attitude and some risks for HCWs in the health facilities in Cameroon. The present results were used to identify the following conclusive remarks. Though the HIV prevalence in HCWs 2.61% is lower than in the general population in Cameroon estimated at 4.3%, there is a high risk of infection for this category of person as we observed a relatively high percentage of AEB 44.62% amongst HCWS with an HIV prevalence of 4.20%. We also demonstrated that the unwillingness to get tested for HIV and ignorance of their HIV status is a major risk factor for the HCWs and the patients they cater for. Lack of appropriate use of PPE by HCWs could explain the rate of AEB amongst HCWs. There is thus, a need for capacity strengthening and provision of psychosocial support to HCWs. The prevalence of HCWs though lower than the national prevalence, translates a high disease burden of HIV and AIDS infection amongst HCWs. We believe this prevalence was minored, as there was uncooperative attitude of health workers and unwillingness to get tested for HIV.

### What is known about this topic

- Studies have evaluated the prevalence of HIV amongst Health Care Workers (HCWs) and reported a relatively high prevalence of HIV and AIDS amongst HCWs;
- HCWs are a special population to be considered in the fight against HIV and AIDS as they are in contact with patients and constantly subjected to occupational exposures in the course of their duty.

### What this study adds

- The prevalence of HIV infection within health workers in Cameroon is lower in HCWs than that in general population;
- AEB constitutes a risk of HIV infection within health workers in Cameroon;
- The prevalence of HIV is high in private than that in public HFs - The HIV screening history is to be consider in relation to HIV testing.
